# Grey matter OPCs are less mature and less sensitive to IFNγ than white matter OPCs: consequences for remyelination

**DOI:** 10.1038/s41598-018-19934-6

**Published:** 2018-02-01

**Authors:** Dennis H. Lentferink, Jacomien M. Jongsma, Inge Werkman, Wia Baron

**Affiliations:** Department of Cell Biology, University of Groningen, University Medical Center Groningen, A. Deusinglaan 1, 9713 AV Groningen, The Netherlands

## Abstract

Multiple sclerosis (MS) is a chronic inflammatory disease characterized by the formation of demyelinated lesions in the central nervous system. At later stages of the disease repair in the form of remyelination often fails, which leads to axonal degeneration and neurological disability. For the regeneration of myelin, oligodendrocyte progenitor cells (OPCs) have to migrate, proliferate and differentiate into remyelinating oligodendrocytes. Remyelination occurs faster and is more extensive in grey matter (GM) lesions than in white matter (WM) lesions. Here, we examined differences in neonatal OPCs from GM (gmOPCs) and WM (wmOPCs), both intrinsically and in response to environmental (injury) signals. We show that gmOPCs are less mature than wmOPCs, both on morphological and on gene-expression level. Additionally, gmOPCs proliferate more and differentiate slower than wmOPCs. When exposed to astrocyte-secreted signals wmOPC, but not gmOPC, migration decreases. In addition, wmOPCs are more sensitive to the detrimental effects of IFNγ treatment on proliferation, differentiation, and process arborisation, which is potentiated by TNFα. Our results demonstrate that OPCs from GM and WM differ both intrinsically and in response to their environment, which may contribute to the difference in remyelination efficiency between GM and WM MS lesions.

## Introduction

Multiple sclerosis (MS) is a chronic inflammatory disease in which demyelinated lesions are present both in the grey (GM) and white matter (WM) of the central nervous system. MS presents as a relapsing remitting (RRMS) or progressive disease course. Demyelinated axons can be remyelinated by endogenous oligodendrocyte progenitor cells (OPCs), which involves their activation and migration to the demyelinated lesion where they will proliferate and mature into remyelinating oligodendrocytes (OLGs)^1^. However, remyelination capacity decreases with age^2,3^ and varies significantly between patients^4^. Post mortem analyses of MS lesions revealed that in 30% of the lesions remyelination fails by a malfunction in OPC recruitment, and in 70% by an inhibition of OPC differentiation^5–7^. Since myelin regeneration is essential for axonal survival, this in turn leads to secondary neurodegeneration, which is most profound in progressive MS^8^. While there are some immunomodulatory drugs available that alter RRMS disease course by reducing the number and severity of relapses, no effective treatments are available for progressive stages. A therapy aimed at improving remyelination capacity might prove beneficial for MS patients. For the development of such a drug, a thorough understanding of OPCs and the process of remyelination is imperative.

In MS lesions^7,9,10^ and upon toxin-induced demyelination^11^, remyelination is more efficient in the GM than in the WM. This may at least partially be due to a higher OPC density in GM lesions^7^, and micro-environmental factors influencing OPC differentiation like spatial differences in inflammatory signals^12–16^, extracellular matrix composition^7^ and differences in the spatial and temporal expression of growth factors^17^. However, local differences in remyelination efficiency might also be explained by regional heterogeneity in OPCs. Indeed, *in vivo*, OPCs in the WM (wmOPCs) produce mature myelinating OLGs more efficiently than OPCs in the GM (gmOPCs), which proliferate slower and produce fewer mature cells^18–21^. Additionally, OPC density is higher in WM than in GM^18,19^, which may be a result of the difference in proliferation rate^18^. When observed in their own respective environments, the expansion phase of gmOLGs in development is much longer compared to wmOLGs, and OLG turnover is higher in human GM than in human WM^22^. When OPCs from GM (cortex) and WM (corpus callosum) are homo- and heterotopically transplanted, wmOPCs differentiate equally well into mature OLGs in both healthy GM and WM upon transplantation. In contrast, gmOPCs remain more immature irrespective of the environment^23^, indicating intrinsic differences between regional OPCs.

Here, we aimed to address intrinsic differences in functional behaviour of gmOPCs and wmOPCs that are relevant for remyelination, including migration, proliferation, survival, differentiation and myelin membrane formation. In addition, we assessed whether regional OPCs differentially respond to environmental signals that are present in healthy tissue, such as factors secreted by astrocytes, and in demyelinated (MS) lesions, such as the pro-inflammatory cytokines tumour necrosis factor-α (TNFα) and interferon-γ (IFNγ)^24^. Our findings revealed that gmOPCs are less mature and wmOPCs are more susceptible to IFNγ-mediated inhibition of OPC proliferation, differentiation and process arborisation. These intrinsic and functional differences may contribute to the observed increased remyelination efficiency of demyelinated GM lesions compared to WM lesions in physiological and pathological conditions, i.e., MS.

## Results

### GmOPCs are morphologically less mature than wmOPCs

**Figure 1:**
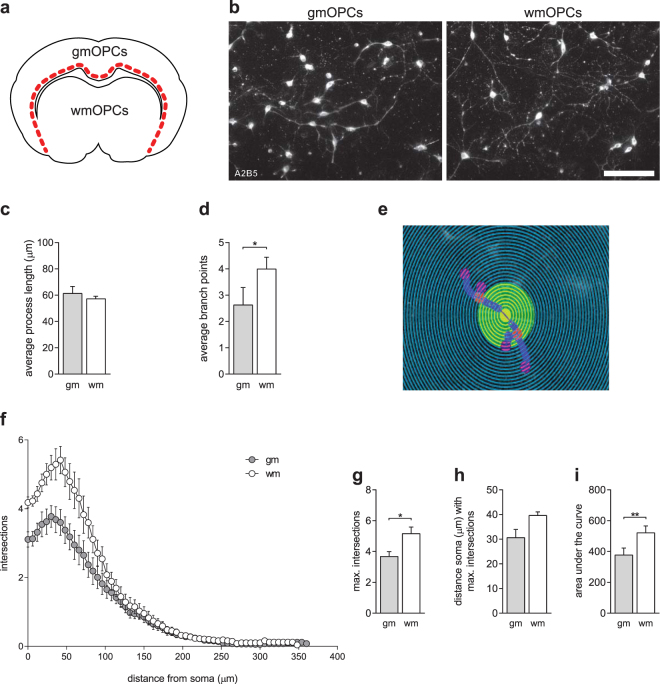
GmOPCs are morphologically less mature than wmOPCs. Oligodendrocyte progenitor cells (OPCs) isolated from the cortex (gmOPCs) and non-cortex (wmOPCs) of neonatal rat forebrains were cultured in the presence of PDGF-AA and FGF-2 for 48 hours. (**a**) Schematic representation of dissected areas of neonatal rat forebrains to obtain gmOPCs and wmOPCs (**b**) OPCs stained with the OPC cell surface marker antibody A2B5. Representative images are shown. (**c–h**) Analysis of the morphology of (**b**) using Sholl analysis of gmOPCs and wmOPCs of the same batch. The process length (**c**), the number of branch points (**d**), the number of processes that intersect with the concentric circles of the Sholl analysis as a function of the distance from the soma (**f**), maximum intersections (**g**), distance of the soma with the maximum number of branch points (**h**) and area under the curve (**i**) are shown. A representative image of the Sholl analysis is shown in (**e**). Note that while the process length is similar, wmOPCs have more branch points, a larger maximum of intersections and total area under the Sholl curve, indicating a more complex branched phenotype than gmOPCs. Bars represent mean process length (**c**), branch points (**d**), maximum intersections (**g**), distance of the soma with the maximum number of branch points (**h**) and area under the curve (**i**) of five independent experiments (24–26 cells analysed per independent experiment). Error bars show the standard error of the mean. Statistical analyses were performed using a paired two-sided t-test (*p < 0.05, **p < 0.01). Scale bar is 50 µm.

To bypass the effect of differences in regional signalling factors that may obscure intrinsic differences of OPCs, OPCs from the cerebral cortex (GM, referred to as gmOPCs) and non-cortical parts (mainly WM, referred to as wmOPCs) of neonatal rat forebrains were isolated (Fig. 1a) and cultured for 12 days with their respective astrocytes and microglia. To maintain OPCs, i.e., to prevent differentiation, cells were cultured in the presence of PDGF-AA and FGF-2. As OPC morphology represents their maturation stage, the surface of cultured OPCs was immunolabelled with the anti-ganglioside antibody A2B5, an OPC-specific surface marker (Fig. 1b). To examine the complexity of OPC morphology, cellular processes were traced and subsequently analysed using Sholl analysis, which quantifies the number of process intersections against the radial distance from the soma center^25,26^. While gmOPCs and wmOPCs had a similar average process length (Fig. 1c; respectively 61.3 ± 5.3 µm and 57.2 ± 2.0 µm, p = 0.363), the average number of branch points was lower in gmOPCs than in wmOPCs (Fig. 1d, respectively 2.6 ± 0.7 and 4.0 ± 0.4, p = 0.026). Sholl analysis (Fig. 1e,f) showed a higher maximum number in process intersections of wmOPCs compared to gmOPCs (Fig. 1g, respectively 5.2 ± 0.4 and 3.7 ± 0.3, p = 0.021), while the distance from the soma with the maximum number of intersections was similar (Fig. 1h, respectively 39.6 ± 1.5 and 30.6 ± 3.3, p = 0.113). When plotting the number of intersections against the distance of the soma, the area under the curve (AUC) was larger for wmOPCs than for gmOPCs (Fig. 1i, respectively 521.2 ± 44.3 and 377.3 ± 44.9, p = 0.004), indicating a more complex morphology of wmOPCs.

### GmOPCs are less mature than wmOPCs on gene expression level

**Figure 2:**
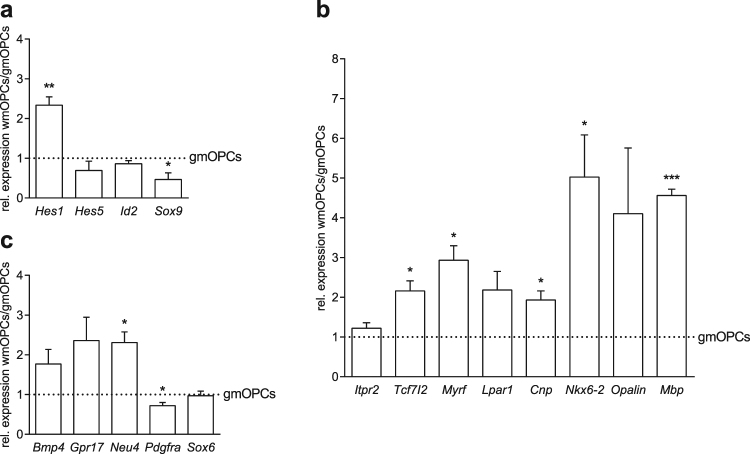
GmOPCs are less mature than wmOPCs on gene expression level. Oligodendrocyte progenitor cells (OPCs) isolated from the cortex (gmOPCs) and non-cortex (wmOPCs) of neonatal rat forebrains were cultured in the presence of PDGF-AA and FGF-2 for 48 hours. OPC were subjected to qPCR analysis of markers that (i) inhibit OPC differentiation (**a**, immature markers), (ii) are associated with myelination (**b**, mature markers, in ascending order of maturity) and (iii) are enriched in differentiation committed OPCs (**c**, COP markers). *Hmbs* was used as reference gene; the reference gene *Eef1a1* showed similar results (data not shown). Note that the mRNA expression levels of the more mature OPC markers are increased in wmOPCs compared to gmOPCs. Bars represent mean expression levels relative to gmOPCs, which were set at 1 for each independent experiment (horizontal line). Error bars show the standard error of the mean. Statistical analyses were performed using a one-sample t-test (*p < 0.05, **p < 0.01, ***p < 0.001, n = 4).

To examine whether this difference in morphological maturity is also reflected at the gene expression level, we next determined the mRNA expression level of genes that specify the maturation state of OPCs. The mRNA expression level of the transcription factor *Hes1*, an inhibitor of myelination^27^ was higher in wmOPCs than in gmOPCs (Fig. 2a, 2.34 ± 0.21 fold change, p = 0.008), whereas the immature OPC transcription factor *Sox9*^28^ was lower in wmOPCs than in gmOPCs (Fig. 2a, 0.47 ± 0.17 fold change, p = 0.048). The expression levels of *Id2* and *Hes5*, transcription factors inhibiting myelination^29–31^, were similar between gmOPCs and wmOPCs [Fig. 2a, respectively 0.86 ± 0.08 (p = 0.172) and 0.69 ± 0.23 (p = 0.279) fold change]. Intriguingly, the mRNA levels of several genes that are associated with myelination were significantly higher in wmOPCs than in gmOPCs (Fig. 2b). These include the transcription factors *Tcf7l2*^32^ (Fig. 2b, 2.16 ± 0.25 fold change, p = 0.020), *Myrf* ^33^ (Fig. 2b, 2.93 ± 0.37 fold change, p = 0.013) and *Nkx6-2*^34^ (Fig. 2b, 5.03 ± 1.06 fold change, p = 0.032) and the myelin proteins *Cnp*^30^ (Fig. 2b, 1.93 ± 0.23 fold change, p = 0.026) and *Mbp*^30^ (Fig. 2b, 4.56 ± 0.16 fold change, p = 0.0002). The mRNA expression of OPC maturity markers *Itpr2*^35^, *Lpar1*^36^ and *Opalin*^30^ did not differ significantly between gmOPCs and wmOPCs [Fig. 2b, respectively 1.22 ± 0.14 (p = 0.200), 2.18 ± 0.47 (p = 0.086) and 4.10 ± 1.65 (p = 0.157) fold change]. These findings indicate that gmOPCs are less mature than wmOPCs, while the higher levels of *Hes1* in the more mature wmOPCs may prevent wmOPC differentiation. Recently, a single cell analysis study has identified the so-called differentiation committed oligodendrocyte progenitor cells (COPs), which represent more mature OPCs^35^. To assess whether wmOPCs may resemble COPs, we next investigated genes that are highly expressed (*Neu4*, *Bmp4*, *Gpr1*7 and *Sox6*) or downregulated (*Pdgfra*) in COPs. The mRNA expression level of *Neu4* was approx. 2-fold higher in wmOPCs (Fig. 2c, 2.31 ± 0.27 fold change, p = 0.016), while *Pdgfra* mRNA levels were significantly lower in wmOPCs than in gmOPCs (Fig. 2c, 0.72 ± 0.08 fold change, p = 0.041). The mRNA levels of *Bmp4* and *Gpr17*, COP-related genes involved in keeping OPCs undifferentiated^35^, were also approx. 2-fold higher in wmOPCs than in gmOPCs, albeit not significant [Fig. 2c, respectively 1.77 ± 0.37 (p = 0.127), and 2.36 ± 0.58 (p = 0.103) fold change]. Hence, *in vitro* gmOPCs were less mature than wmOPCs both morphologically and at the gene expression level of OPC differentiation-associated genes. To assess whether these differences in maturity of gmOPCs and wmOPCs are translated into functional differences, we next examined cell behavioural processes that are relevant to remyelination.

### GmOPCs proliferate more and differentiate slower than wmOPCs

**Figure 3:**
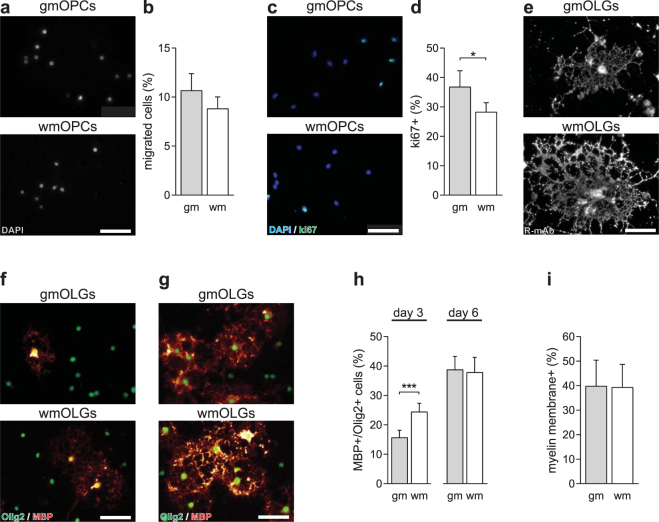
WmOPCs proliferate less and differentiate faster than gmOPCs. Oligodendrocyte progenitor cells (OPCs) isolated from the cortex (gmOPCs) and non-cortex (wmOPCs) of neonatal rat forebrains were cultured for 4 hours (**a,b**) or 48 hours in the presence of PDGF-AA and FGF-2 (**c–i**), followed by differentiation for 3 (immature stage, **e,f** and **h**) or 6 days (mature stage, **g–i)**. (**a**,**b**) OPC migration towards a 10 ng/ml PDGF-AA gradient (4 hours) was determined using a transwell assay. Representative images of migrated DAPI-stained OPCs are shown in **a**; quantitative analyses of the percentage of DAPI-stained migrated OPCs of the total number of plated cells in (**b**) (n = 10), (**c,d**) OPC proliferation was determined by immunocytochemistry for the proliferation marker ki67. Representative images are shown in (**c**), quantitative analyses of the number of ki67-positive of total DAPI-stained cells in (**d**) (n = 16, at least 150 cells analysed per independent experiment). Note the higher percentage of proliferating gmOPCs compared to wmOPCs. (**e–i**) OPC were differentiated for 3 (**e**,**f**, and **h**) and 6 days (**g–i**) and incubated with either (**e**) R-mAb, recognizing GalCer/sulfatide, or (**f–i**) double stained for MBP (red), a mature marker of oligodendrocytes (OLGs) and Olig2 (green), OLG lineage marker. Representative images are shown in (**e**,**f**) and (**g**); quantitative analyses of the number of MBP-positive OLGs of total Olig2-positive cells in (**h**) **(**n = 8 for 3 days, n = 10 for 6 days, at least 150 cells analysed per independent experiment) and the number of MBP-positive cells that elaborate myelin membranes in (**I**) (n = 10, 6 days). Note that after 3 days of differentiation wmOLGs are larger and morphologically more complex than gmOPCs. In addition, wmOPCs show an accelerated differentiation, while the number of MBP-positive cells bearing myelin membranes at day 6 is similar. Bars represent means. Error bars show the standard error of the mean. Statistical analyses were performed using a paired two-sided t-test (*p < 0.05, ***p < 0.001). Scale bar is 50 µm.

Upon demyelination one of the first events is the migration of activated adjacent OPCs to the lesioned area. To assess whether gmOPCs and wmOPCs differ in their migratory capacity, gmOPCs and wmOPCs were cultured on a porous membrane, and cellular migration towards a PDGF-AA gradient was examined. The percentage of gmOPCs that have migrated in 4 hours across the transwell membrane was similar to the percentage of migrated wmOPCs (Fig. 3a,b, respectively 10.7 ± 1.7% and 8.8 ± 1.2%, p = 0.146). In addition, OPCs have to proliferate to obtain sufficient numbers for successful remyelination. After a 48-hour exposure to the mitogens PDGF-AA and FGF-2, the percentage of cells positive for the proliferation marker ki67 was higher in gmOPCs than in wmOPCs (Fig. 3c,d, respectively 36.8 ± 5.6% and 28.3 ± 3.2%, p = 0.048). The final step in remyelination is the differentiation of OPCs towards mature myelinating OLGs. Fluorescent imaging of GalCer/sulfatide by R-mAb showed that wmOPCs that maturated into wmOLGs were larger than gmOPCs that maturated into gmOLGs (Fig. 3e) corroborating a recent finding that OLGs show regional heterogeneity in morphology^37^. Immunofluorescent labelling of MBP, a marker for mature OLGs, showed that significantly more wmOPCs expressed MBP after 3 days of differentiation than gmOPCs (Fig. 3f,h, respectively 24.4 ± 3.0%and 15.7 ± 2.5%, p = 0.0003). However, after 6 days of differentiation the percentage of MBP-positive cells of gmOPCs and wmOPCs was similar (Fig. 3g,h, respectively 38.8 ± 4.5% and 37.8 ± 5.2%, p = 0.862). A read-out parameter for ‘myelination’ in OLG monocultures is the number of cells that elaborate MBP-positive myelin membranes of total MBP-positive cells. GmOLGs and wmOLGs hardly differed in their ability to form myelin membranes *in vitro* (Fig. 3g,i, respectively 39.8 ± 10.6% and 39.3 ± 9.4%, p = 0.926). As the percentage of MBP-positive cells reaches a maximum at day 6, these findings indicate that the differentiation of wmOPCs was accelerated *in vitro*. Hence, *in vitro* gmOPCs proliferated more, while wmOPCs differentiated faster and elaborated more extensive process networks. Next to intrinsic differences in functional behaviour, a distinct response of gmOPCs and wmOPCs towards micro-environmental signals may also contribute to differences in (re)myelination efficiency.

### WmOPCs migrate less in response to astrocyte conditioned medium than gmOPCs

**Figure 4:**
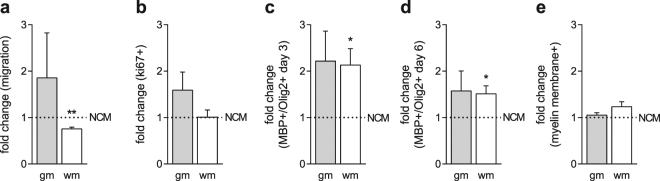
WmOPCs migrate less in response to astrocyte secreted factors than gmOPCs. Oligodendrocyte progenitor cells (OPCs) isolated from the cortex (gmOPCs) and non-cortex (wmOPCs) of neonatal rat forebrains were treated with non-conditioned medium (NCM) or cultured in the presence of astrocyte conditioned medium (ACM) at the indicated time points for the duration of the experiment. (**a**) OPC migration towards a 10 ng/ml PDGF-AA gradient (4 hours) was determined using a transwell assay (n = 5). Note that exposure to ACM decreased the migration of wmOPCs compared to NCM treatment, while gmOPC migration tends to increase upon ACM exposure. (**b**) Following 24 hours in culture, OPCs were exposed to NCM and ACM for 24 hours in the presence of PDGF-AA and FGF-2. OPC proliferation was determined by immunocytochemistry for the proliferation marker ki67 (n = 7). (**c–e**) OPCs were differentiated in NCM or ACM for 3 (**c**) and 6 days (**d,e**) and subjected to a double staining for MBP (red), a mature marker of oligodendrocytes (OLGs) and Olig2 (green), an oligodendrocyte (OLG) lineage marker (n = 5). Note that ACM increased differentiation (**c,d**), while myelin membrane formation is hardly affected (**e**). Bars represent mean relative to their respective NCM-treated control, which was set at 1 for each independent experiment (horizontal line). Error bars show the standard error of the mean. Statistical analyses were performed using a one-sample t-test (*p < 0.05, **p < 0.01) to test for differences between treatments and their respective control and an unpaired two-sided t-test was used to test whether the response to ACM differed between gmOPCs and wmOPCs (not significant).

Astrocytes are important regulators of OPC behaviour^38,39^. To examine whether gmOPCs and wmOPCs respond differently to astrocyte-derived factors we exposed gmOPCs and wmOPCs to astrocyte conditioned medium (ACM) and determined the effect on OPC migration, proliferation and differentiation. To this end, non-conditioned medium (NCM) and ACM were added to OPCs for the duration of the experiment. Exposing OPCs for 24 hours to ACM impairs wmOPC, but not gmOPC migration [Fig. 4a, respectively 0.76 ± 0.4 (p = 0.003) and 1.86 ± 0.97 (p = 0.427) fold change], while proliferation of gmOPCs and wmOPCs was hardly affected upon ACM exposure [Fig. 4b, respectively 1.59 ± 0.39, (p = 0.181) and 1.01 ± 0.15 (p = 0.952) fold change]. Furthermore, upon ACM exposure the percentage of MBP-expressing cells was increased after 3 days and 6 days of differentiation [respectively Fig. 4c, 2.13 ± 0.35 (p = 0.033) fold change and Fig. 4d, 1.51 ± 0.17 (p = 0.040) fold change]. The effect of ACM exposure on gmOPC differentiation was more variable, although a similar but not significant increase in differentiation was observed [Fig. 4c, 2.22 ± 0.65 (p = 0.134) fold change and Fig. 4d, 1.58 ± 0.43 (p = 0.251) fold change]. This suggests that secreted molecules in ACM per se stimulate OPC differentiation. Myelin membrane formation in both gmOLGs and wmOLGs was hardly if at all altered upon addition of ACM [Fig. 4e, respectively 1.05 ± 0.05 (p = 0.352) and 1.23 ± 0.11 (p = 0.093) fold change]. Hence, wmOPCs were more receptive to astrocyte secreted signals, which alter functional endpoints relevant for myelination, i.e., migration and differentiation. In MS lesions, other factors like the pro-inflammatory cytokines TNFα and IFNγ may influence remyelination capacity of OPCs^24,40–45^. Also, pro-inflammatory cytokines seem to play a role in the pathology of rodent models of MS, including experimental autoimmune encephalomyelitis and cuprizone-induced demyelination^46–48^. Therefore, we next examined the effect of TNFα and IFNγ on gmOPC and wmOPC morphology and behaviour *in vitro*.

### IFNγ increases the process length of gmOPCs and wmOPCs and reduces the number of branch points in wmOPCs

**Figure 5:**
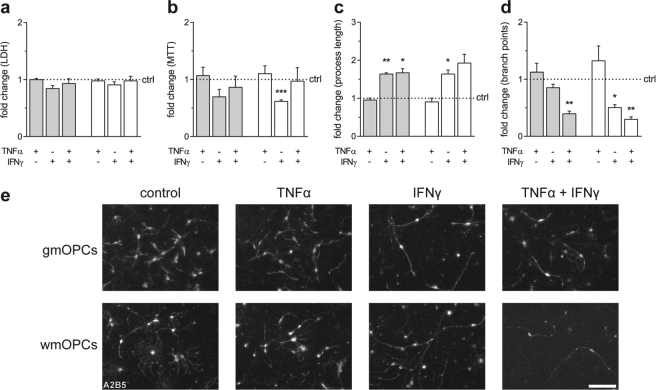
IFNγ increases the process length of gmOPCs and wmOPCs and reduces the number of branch points in wmOPCs. Oligodendrocyte progenitor cells (OPCs) isolated from the cortex (gmOPCs) and non-cortex (wmOPCs) of neonatal rat forebrains were left untreated or treated with 10 ng/ml TNFα, 500 U/ml IFNγ, or a combination of TNFα and IFNγ for 48 hours in the presence of PDGF-AA and FGF-2. (**a**) Cell cytotoxicity as measured with an LDH assay (n = 4). (**b**) Cell viability as measured with MTT reduction (n = 4). Note that IFNγ treatment reduces the MTT reduction in both gmOPCs and wmOPCs compared to their respective untreated control. (**c–e**) OPCs stained with the OPC cell surface marker antibody A2B5. Representative images are shown (**e)**. (**c,d**) Analysis of the morphology of gmOPCs and wmOPCs of the same batch. The process length (**c**, n = 3) and the number of branch points (**d**, n = 3) are shown. Note that IFNγ increases the process length of gmOPCs and wmOPCs (**c**) and reduces the number of branch points in wmOPCs, but not gmOPCs (**d**). When IFNγ is combined with TNFα the number of branch points is decreased in either OPC. Bars represent mean relative to their respective untreated control, which was set at 1 for each independent experiment (horizontal line). Grey bars represent gmOPCs, white bars represent wmOPCs. Error bars show the standard error of the mean. Statistical analyses were performed using column statistics with a one-sample t-test (*p < 0.05, **p < 0.01,***p < 0.001) to test for differences between treatments and their respective control and a one-way ANOVA with a Šidák post-test was used to test whether the response to TNFα, IFNγ and TNFα and IFNγ combined differed between gmOPCs and wmOPCs (not significant). Scale bar is 50 µm.

**Figure 6:**
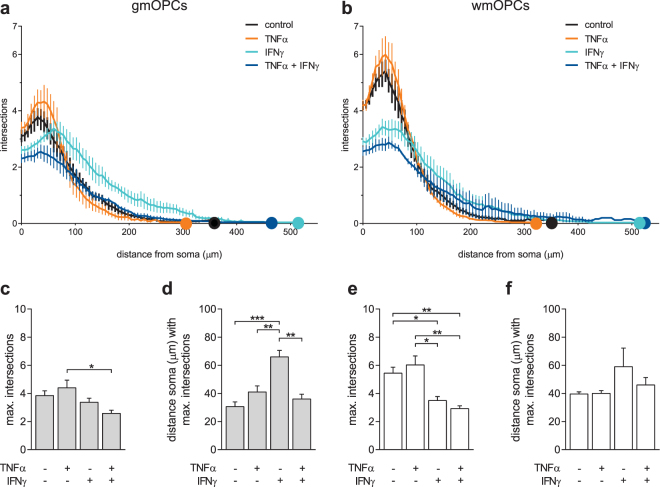
IFNγ decreases the maximum number of intersections in gmOPCs and wmOPCs and increases the distance of the soma with the maximum number of intersections of gmOPCs. Oligodendrocyte progenitor cells (OPCs) isolated from the cortex (gmOPCs) and non-cortex (wmOPCs) of neonatal rat forebrains were left untreated or treated with 10 ng/ml TNFα, 500 U/ml IFNγ, or a combination of TNFα and IFNγ for 48 hours in the presence of PDGF-AA and FGF-2. OPCs were stained for the OPC cell surface marker A2B5 to visualize their morphology (see Fig. 4e). The number of processes that intersect with the concentric circles of Sholl analysis as a function of the distance from the soma (**a**,**b**), maximum intersections (**c**,**e**) and distance of the soma with the maximum number of branch points (**d**,**f**) are shown. Grey bars represent gmOPCs (**a**,**c**,**d**), white bars represent wmOPCs (**b**,**e**,**f**). Error bars show the standard error of the mean. Note that the maximum number of intersections is decreased in wmOPCs upon treatment with IFNγ, and in both gmOPCs and wmOPCs upon treatment with IFNγ combined with TNFα, while the distance of the soma with the maximum number of intersections is increased upon IFNγ treatment in gmOPCs. Statistical analyses were performed using a one-way ANOVA with a Tukey’s post-test (*p < 0.05, **p < 0.01, ***p < 0.001) to test for differences between treatments and their respective control. Scale bar is 50 µm.

Inflammation is a hallmark of MS, and pro-inflammatory cytokines TNFα and IFNγ have been demonstrated to play a role in the disease^45,49–51^. OPCs in an MS lesion environment have been exposed to these cytokines, which could result in an altered remyelination capacity. We asked whether exposure of OPCs to these cytokines *in vitro* might affect functional endpoints relevant for remyelination. First, to assess the cytotoxicity of cytokine treatments on OPCs, LDH and MTT-reduction assays were performed upon 48-hour treatment. Exposure to TNFα, or IFNγ hardly if at all induced cytotoxicity in both gmOPC and wmOPCs [Fig. 5a, respectively 0.84 ± 0.05 (p = 0.059) and 0.91 ± 0.05 (p = 0.191) fold change]. However, IFNγ treatment significantly reduced MTT-reduction of wmOPCs (Fig. 5b, 0.62 ± 0.03 fold change, p = 0.001), an effect which is less pronounced and not significant in gmOPCs (Fig. 5b, 0.70 ± 0.31 fold change, p = 0.105), indicating reduced metabolic activity in IFNγ-treated wmOPCs. Consecutively, morphological assays were performed. IFNγ treatment markedly increased the process length of both gmOPCs and wmOPCs [Fig. 5c, respectively 1.64 ± 0.03 (p = 0.002) and 1.64 ± 0.10 (p = 0.025) fold change], whereas exposure to TNFα did not affect process length [Fig. 5c, respectively 0.95 ± 0.05 (p = 0.454) and 0.91 ± 0.10 (p = 0.439) fold change]. TNFα and IFNγ together significantly increased gmOPC process length to a similar extent as IFNγ treatment (Fig. 5c, 1.67 ± 0.10 fold change, p = 0.023). As shown in Fig. 5d, upon IFNγ exposure the number of branch points decreased significantly in wmOPCs (0.50 ± 0.05 fold change, p = 0.010), but not in gmOPCs (0.85 ± 0.06 fold change, p = 0.139). Remarkably, the effect of IFNγ on wmOPCs was potentiated upon combined treatment with TNFα (Fig. 5d, 0.30 ± 0.04 fold change, p = 0.004), while exposure to TNFα was seemingly ineffective both in gmOPCs and wmOPCs [Fig. 5d, respectively 1.12 ± 0.15 (p = 0.501) and 1.32 ± 0.26 (p = 0.340) fold change]. Sholl analysis further revealed that IFNγ treatment reduced the maximum number of process intersections in wmOPCs compared to untreated wmOPCs (Fig. 6b,e, respectively 3.49 ± 0.29 and 5.16 ± 0.42, p = 0.004), but not in gmOPCs (Fig. 6a,c, respectively 3.37 ± 0.29 and 3.84 ± 0.34, p = 0.786). However, in gmOPCs the maximum number of intersections shifted towards a higher distance from the soma upon exposure to IFNγ (Fig. 6a,d, respectively 66.0 ± 4.6 and 30.6 ± 3.3, p = 0.0003), which was not evident in wmOPCs (Fig. 6b,f, respectively 59.0 ± 13.2 and 39.6 ± 1.5, p = 0.152). Remarkably, combined treatment of IFNγ with TNFα counteracted the effect of IFNγ in gmOPCs, i.e., the distance of the soma with the maximum number of intersections was similar to untreated control and TNFα-treated gmOPCs (Fig. 6a,d, 30.6 ± 3.3 and 36.0 ± 3.5, p = 0.270). Hence, these findings indicate that IFNγ treatment reduces OPC process arborisation, i.e., OPCs appear morphologically less mature upon IFNγ treatment, which was more pronounced in wmOPCs than gmOPCs.

### WmOPCs are more sensitive to TNFα- and IFNγ-mediated inhibition of proliferation than gmOPCs

**Figure 7:**
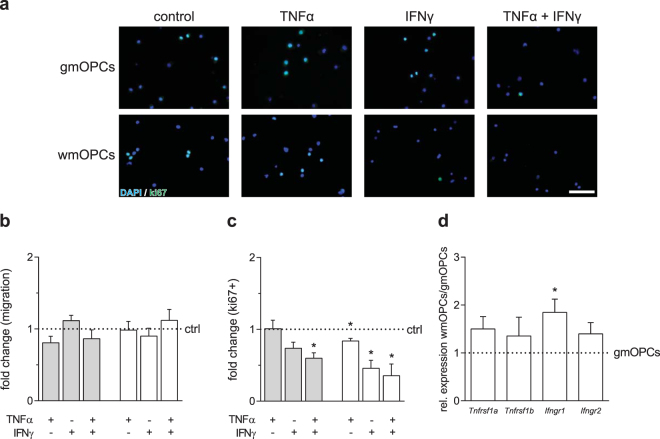
WmOPCs are more sensitive to TNFα- and IFNγ-mediated inhibition of proliferation than gmOPCs. Oligodendrocyte progenitor cells (OPCs) isolated from the cortex (gmOPCs) and non-cortex (wmOPCs) of neonatal rat forebrains were left untreated or treated with 10 ng/ml TNFα, 500 U/ml IFNγ, or a combination of TNFα and IFNγ for 48 hours in the presence of PDGF-AA and FGF-2. (**a,c**) OPC proliferation was determined by immunocytochemistry for the proliferation marker ki67. Representative images are shown in (**a**); quantitative analysis of the number of ki67-positive of total DAPI-stained cells in (**c**) (n = 4, at least 150 cells analysed per independent experiment). (**b**) OPC migration towards a 10 ng/ml PDGF-AA gradient (4 hours) was determined using a transwell assay (n = 5). Grey bars represent gmOPCs white bars represent wmOPCs (**b**,**c**). Note that both exposure to TNFα and IFNγ decreased wmOPC proliferation, while IFNγ, but not TNFα, decreased gmOPC proliferation. (**d**) mRNA expression levels of *Tnfrs1a*, *Tnfrs1b*, *Ifngr1* and *Ifngr2. Hmbs* was used as reference gene; the reference gene *Eef1a1* showed similar results (data not shown). Note that *Ifngr1* expression levels are elevated in wmOPCs compared to gmOPCs. Bars represent mean relative to their respective untreated control, which was set at 1 for each independent experiment (horizontal line). Error bars show the standard error of the mean. Statistical analyses were performed using a one-sample t-test (*p < 0.05) to test for differences between treatments and their respective control and a one-way ANOVA with a Šidák post-test was used to test whether the response to TNFα, IFNγ and TNFα and IFNγ combined differed between gmOPCs and wmOPCs (not significant). Scale bar is 50 µm.

To examine the effect of pro-inflammatory cytokines on cell behaviour aspects that are relevant to OPC recruitment, we next examined the effect of TNFα and IFNγ on gmOPC and wmOPC migration and proliferation compared to their respective untreated control OPCs. Upon exposure of TNFα, IFNγ or a combination of TNFα and IFNγ, the number of migrating cells was similar in gmOPCs [Fig. 7b, respectively, 0.81 ± 0.09 (p = 0.100), 0.90 ± 0.11 (p = 0.418) and 0.86 ± 0.12 (p = 0.334) fold change] and wmOPCs [Fig. 7b, respectively, 0.98 ± 0.12 (p = 0.904), 1.12 ± 0.08 (p = 0.202) and 1.12 ± 0.08 (p = 0.202) fold change]. Exposure to TNFα resulted in a decrease in proliferation in wmOPCs (Fig. 7a,c, 0.84 ± 0.04 fold change, p = 0.021), but not in gmOPCs (Fig. 7a,c, 1.01 ± 0.12 fold change, p = 0.945). Similarly, exposure to IFNγ significantly decreased wmOPC, but not gmOPC proliferation [Fig. 7a,c, respectively 0.46 ± 0.12 (p = 0.017) and 0.74 ± 0.08 (p = 0.051) fold change]. Combined treatment of TNFα and IFNγ synergized in wmOPCs, resulting in a further decrease of proliferation (Fig. 7a,c, 0.35 ± 0.16 fold change, p = 0.028). GmOPC proliferation also decreased upon exposure to both TNFα and IFNγ (Fig. 7a,c, 0.60 ± 0.07 fold change, p = 0.013). Hence, these data indicate that wmOPCs were more sensitive to TNFα- and IFNγ-mediated inhibition of proliferation than gmOPCs. To examine whether the increased sensitivity of wmOPCs was mediated via an increased expression of the TNFα and/or IFNγ receptor, qPCR analysis was performed. The mRNA level of the IFNγ receptor *Ifngr1*, but not *Ifngr2*, was higher in wmOPCs than in gmOPCs [Fig. 7d, respectively 1.85 ± 0.28 (p = 0.038) and 1.40 ± 0.24 (p = 0.163) fold change]. The mRNA levels of the receptors for TNFα (*Tnfrsf1a and Tnfrsf1b*) did not significantly differ between wmOPCs and gmOPCs [Fig. 7d, respectively 1.50 ± 0.26 (p = 0.123) and 1.35 ± 0.39 (p = 0.421) fold change].

### IFNγ delays wmOPC, but not gmOPC differentiation

**Figure 8:**
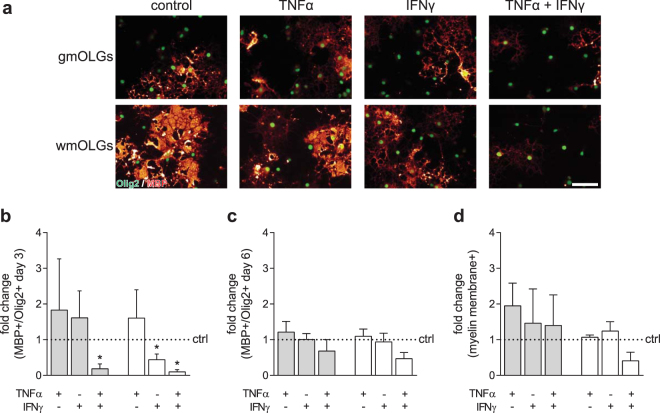
Exposure to IFNγ delays wmOPC, but not gmOPC differentiation. Oligodendrocyte progenitor cells (OPCs) isolated from the cortex (gmOPCs) and non-cortex (wmOPCs) of neonatal rat forebrains were left untreated or treated with 10 ng/ml TNFα, 500 U/ml IFNγ, or a combination of TNFα and IFNγ for 48 hours in the presence of PDGF-AA and FGF-2, followed by differentiation in the absence of cytokines. (**a–d**) OPC differentiation was determined at 3 (**b**) and 6 days (**a,c,d**) of differentiation using double staining for MBP (red), a mature marker of oligodendrocytes (OLGs) and Olig2 (green), an OLG lineage marker. Representative images at 6 days of differentiation are shown in (**a**); quantitative analyses of the number of MBP-positive cells of total Olig2-positive cells in (**b**) **(**3 days, n = 4) and (**c**) (6 days, n = 4) and the number of MBP-positive cells that elaborate myelin membranes in (**d**) (6 days, n = 4). Note that brief exposure to IFNγ at the OPC stage delays the differentiation of wmOPCs, but not of gmOPCs, while combined treatment with TNFα and IFNγ inhibited differentiation of either OPC. Grey bars represent gmOPCs, white bars represent wmOPCs (**b**,**c**,**d**). Error bars show the standard error of the mean. Bars represent mean relative to their respective untreated control, which was set at 1 for each independent experiment (horizontal line). Statistical analyses were performed using a one-sample t-test (*p < 0.05) to test for differences between treatments and their respective control and a one-way ANOVA with a Šidák post-test was used to test whether the response to TNFα, IFNγ and TNFα and IFNγ combined differed between gmOPCs and wmOPCs (not significant). Scale bar is 50 µm.

Upon toxin-induced demyelination, and likely also in MS lesions, OPCs are only transiently exposed to pro-inflammatory cytokines. To mimic the effect of this transient exposure to inflammatory signals, OPCs were treated with TNFα, IFNγ or a combination of TNFα and IFNγ for 48 hours, after which OPCs were allowed to differentiate in the *absence* of cytokines. Upon 3 days of differentiation, a brief exposure to IFNγ at the OPC stage decreased the percentage of MBP-positive wmOLGs, but not of gmOLGs [Fig. 8a,b, respectively 0.44 ± 0.16 (p = 0.039) and 1.61 ± 0.76 (p = 0.477) fold change]. In contrast, TNFα hardly if at all changed gmOPC and wmOPC differentiation [Fig. 8a,b, respectively 1.83 ± 1.44 (p = 0.604) and 1.61 ± 0.79 (p = 0.502) fold change]. Remarkably, exposure to both TNFα and IFNγ drastically decreased both gmOPC and wmOPC differentiation [Fig. 8a,b, respectively 0.19 ± 0.14 (p = 0.009) and 0.10 ± 0.06 (p = 0.001) fold change]. At 6 days of differentiation the decrease in gmOPC differentiation upon treatment with IFNγ was diminished (Fig. 8a,c, 0.94 ± 0.24 fold change, p = 0.818), while a slight but not significant decrease in wmOPC differentiation was still apparent upon TNFα and IFNγ exposure (Fig. 8a,c, 0.47 ± 0.17 fold change, p = 0.055). The percentage of MBP-positive OLGs that form myelin membranes at 6 days of differentiation was hardly affected when gmOPCs and wmOPCs were transiently exposed to the pro-inflammatory cytokines [Fig. 8a,d, respectively 1.95 ± 0.64 (p = 0.232) and 1.07 ± 0.06 (p = 0.351) fold change upon TNFα exposure; 1.46 ± 0.96 (p = 0.663) and 1.24 ± 0.27 (p = 0.432) fold change upon IFNγ exposure; 1.40 ± 0.86 (p = 0.675) and 0.41 ± 0.24 (p = 0.090) fold change upon combined TNFα and IFNγ exposure]. Note that after 6 days of differentiation wmOLGs produced more elaborate myelin membranes than gmOLGs, consistent with the more elaborated network at day 3 of differentiation (Fig. 8a cf Fig. 3e). Hence, brief exposure to IFNγ at the OPC stage delays wmOPC, but not gmOPC differentiation *in vitro* and transient exposure to a combination of TNFα and IFNγ may perturb wmOPC differentiation.

## Discussion

Remyelination at physiological conditions and in MS is more extensive in GM lesions than in lesions of the WM^7,9–11^. Here, we aimed to unravel whether inherent differences in gmOPC and wmOPC behaviour, including their response to environmental (injury) signals, contribute to regional differences in remyelination efficiency. Our *in vitro* findings -based on morphology, proliferation and migration capacity, differentiation kinetics and expression of myelination-associated genes- revealed that neonatal gmOPCs are less mature than neonatal wmOPCs. In addition, wmOPCs were less migratory upon addition of astrocyte secreted factors than gmOPCs, and wmOPCs were also more sensitive to IFNγ-mediated inhibition of proliferation and differentiation than gmOPCs, an effect that was potentiated by TNFα. Given that OPCs revert to a more immature stage upon demyelination^52^, gmOPCs may have evolved to be better equipped for remyelination than wmOPCs, i.e., gmOPCs are more proliferative, less mature, and less responsive to astrocyte-derived factors that affect recruitment and less susceptible to inflammatory mediators than wmOPCs. The more matured stage of wmOPCs may provide an advantage in developmental myelination and myelin remodelling.

Remarkably, most *in vitro* studies have been performed with GM (cortical) OPCs, whereas remyelination and myelination *in vivo* is usually examined in WM areas. Our detailed *in vitro* comparison between neonatal gmOPCs and wmOPCs allowed us to characterize inherent differences between and responsiveness of regional OPCs without the interference of spatial and interacting environmental cues. *In vitro*, gmOPCs were less branched and had a higher capacity to proliferate in response to PDGF-AA and FGF-2 than wmOPCs. Indeed, relatively more OPCs are present in GM MS lesions, while endogenous remyelination in WM MS lesions is hampered by the recruitment of OPCs to the lesion site^7,10,53^. The *in vitro* differentiation of wmOPCs was accelerated compared to gmOPC differentiation. Previous fate mapping studies during CNS development showed that wmOPCs produce more mature myelinating OLGs, while gmOPCs remain immature^18^. Similarly, gmOPC, but not wmOPC maturation is retained when transplanted to either a healthy GM or WM area of the adult mouse brain^23^. Hence, while gmOPCs differentiate *in vitro* eventually to a similar extent as wmOPCs, *in vivo* gmOPCs remain immature irrespective of their environment. The relative maturity of wmOPCs, as evident by a more complex morphology and an increase in the level of genes that are associated with OPC maturation may underlie the accelerated maturation of wmOPCs *in vitro*. In the adult brain a continuum from OPCs to mature OLGs exists, and using a selection of genes, including genes that mark the so-called COPs, i.e., differentiation committed more mature OPCs, we show here that wmOPCs *in vitro* may resemble COPs more than gmOPCs. Indeed, COPs are hardly present in the GM sensory cortex, but abundantly present in the corpus callosum (WM)^35^. This indicates that OPCs *in vivo* are also more mature in the WM than in the GM, and wmOPCs may have started to exit the cell cycle and progressed to these committed OPCs. Of interest in this respect is that the mRNA level of the transcription factor *Hes1*, a negative regulator of myelination was higher in wmOPCs than in gmOPCs, which may keep the more mature and less proliferative wmOPCs undifferentiated in the presence of PDGF-AA and FGF-2. Indeed, it has been reported that combined exposure to these mitogens induces *Hes1* expression in OPCs^27^, which may be more potent in wmOPCs. Also, wmOPCs have reduced *Pdgfra* mRNA levels compared to gmOPCs, which may explain why wmOPCs proliferated less in response to PDGF-AA and FGF-2 than gmOPCs.

OPC migration, proliferation and differentiation are critical for successful remyelination. While the immaturity and the slower differentiation kinetics of gmOPCs seem disadvantageous to remyelination, the opposite may be true. Adult OPCs that are activated upon demyelination return first to a more immature, neonatal-like state and this ‘dedifferentiation’ enhances their migratory capacities *in vitro*^52^. Also, upon chronic cuprizone-induced demyelination, remyelination is faster in the cerebral cortex than in the corpus callosum^11^. In contrast, a recent genetic fate mapping study demonstrate that OPC maturation upon acute cuprizone-mediated demyelination occurs slower in the cingulate cortex and hippocampus than in the corpus callosum^54^, indicating that also within GM areas OPC maturation and remyelination efficiency are heterogeneous. OPCs in the GM and WM are surrounded by different cellular and molecular environments and therefore influenced by distinct regional cues that may orchestrate OPC maturity. In addition, the origin of OPCs may signify functional differences in OPCs in GM and WM. The brain is populated by three sequential OPC waves that are generated from different regions of the forebrain ventricular zone^55^. At postnatal day 2 the third wave has populated the cortex, but not yet WM areas. This may account for intrinsic differences in neonatal gmOPCs and wmOPCs.

Differences in maturation stage of gmOPCs and wmOPCs may also explain their differential responsiveness to secreted factors from normal astrocytes. In response to ACM, wmOPC migration was decreased and wmOPC differentiation was increased compared to NCM-exposed control wmOPCs, while gmOPCs were less responsive to ACM than wmOPCs. It should be noted that in the present study, ACM was derived from non-activated astrocytes, while in MS lesions astrocytes become reactive. Of interest, two distinct subsets of reactive astrocytes have been described, neurotoxic A1 and neurotrophic A2 astrocytes^56^. In contrast to the conditioned medium of normal astrocytes, neurotoxic A1 astrocytes inhibit gmOPC proliferation and differentiation^56^, while ACM of LPS-activated astrocytes also inhibit gmOPC differentiation via secreted TNFα^57^. Whether secreted factors from reactive astrocytes in the inflammatory MS lesion environment differentially affect gmOPC and wmOPC behaviour and whether this is of relevance to remyelination (failure) remains to be determined.

Upon demyelination OPCs face and respond to inflammatory mediators. The transient expression of the pro-inflammatory cytokines TNFα and IFNγ coincides with demyelination^48,58,59^, while their accumulation in MS lesions is suggested to associate with OLG cell death^24,49^. Although TNFα and IFNγ have been described to be cytotoxic to gmOPCs^41,48,60^, in the present study no increase in gmOPC and wmOPC cytotoxicity was found at defined treatment conditions. Remarkably, while TNFα was seemingly ineffective, in IFNγ-treated wmOPCs a decrease in MTT reduction and proliferation was observed, consistent with previous findings in gmOPCs^61^. In addition, upon IFNγ exposure an increase in process length was noticed in both gmOPCs and wmOPCs, while a decrease in process ramification and decelerated differentiation was observed in wmOPCs only. Strikingly, brief and transient exposure of OPCs to IFNγ is sufficient to delay wmOPC differentiation, an effect that was potentiated by TNFα. One of the few *in vitro* studies with wmOPCs shows that continuous exposure to IFNγ perturbs differentiation, while in the current study transient exposure of wmOPCs to IFNγ had a long-term effect on differentiation. Hence, while retaining their ability to differentiate, brief exposure to IFNγ or IFNγ and TNFα of resident OPCs in WM MS lesions may delay their differentiation, which is evidently of relevance for the development of therapeutic strategies aimed at restoring remyelination.

Taken together, neonatal gmOPCs and wmOPCs display their own distinct identity *in vitro*, as, among others, reflected by differences in morphology, maturity and responses to environmental (injury) signals, including pro-inflammatory cytokines. Unravelling the underlying molecular mechanisms is not only crucial for understanding OPC heterogeneity, but also for the development of therapeutic interventions, as distinct strategies may be needed to restore remyelination in GM or WM MS lesions.

## Methods

### Primary cell cultures

#### Oligodendrocyte progenitor cells (OPCs)

Animal protocols were approved by the Institutional Animal Care and Use Committee of the University of Groningen (the Netherlands). All methods were carried out in accordance with national and local experimental animal guidelines and regulations. OPCs were isolated from the neonatal cortex (Fig. 1a, referred to as gmOPCs) and neonatal non-cortical parts (WM tracts including corpus callosum, mixed GM and WM tracts, including hippocampus and thalamus, and deep GM parts, including basal ganglia, Fig. 1a, referred to as wmOPCs) of rat forebrains using a shake-off procedure as described previously^62,63^. A detailed description of the method is provided in the supplementary information. The enriched OPC fraction contained 95–97% OPCs (Olig2-positive), less than 1% microglia (IB4-positive), 1–3% astrocytes (GFAP-positive) and less than 1% neurons (TuJ1-positive) for both gmOPCs and wmOPC cultures. OPCs were cultured on 13-mm poly-L-lysine (PLL, 5 µg/ml)-coated glass slides in 24-well plates unless stated otherwise. With the exception of migration and adhesion assays, cells were plated at a density of 30.000 (GM) or 40.000 (WM) cells per well in defined Sato medium^62^ (see supplementary information). OPCs were synchronized to the bipolar early OPC stage by addition of 10 ng/ml platelet-derived growth factor-AA (PDGF-AA; Peprotech, cat. no. 100–13A) and 10 ng/ml human fibroblast growth factor-2 (FGF-2; Peprotech, cat. no. 100-18B) 1 hour after plating. Where indicated, 1 hour after plating cells were exposed for 48 hours to cytokines TNFα (10 ng/ml) and/or IFNγ (500 U/ml). After 2 days OPCs were allowed to differentiate in Sato medium supplemented with 0.5% fetal bovine serum (FBS) for 3 (immature oligodendrocytes (OLGs)) or 6 days (mature OLGs).

#### Astrocytes

Remaining astrocytes of the mixed glia cell culture flasks (see supplementary information) were passaged once by trypsinization and transferred to 162 cm^2^ flasks and cultured in astrocyte medium (100 U penicillin and streptomycin, 4 mM L-glutamine, 10% heat-inactivated FBS (Bodinco, cat. no. 4005-BDC-0814) in DMEM). The enriched astrocyte fraction yielded a highly pure >97% astrocyte population. Upon reaching confluency, cells were trypsinised and plated in 6-well plates at 1*10^6^ cells per well in astrocyte medium. After one day cells were washed with PBS and cultured for 24 hours in Sato medium. ACM was collected, filtered using a 0.45 µM filter and stored at −20 °C until further use. OPCs were cultured in ACM with a 1:1 ratio with Sato supplemented with 0.5% FCS where indicated.

### Immunocytochemistry

Live cell immunolabelling of Ranscht-mAb (R-mAb; recognizing GalCer/sulfatide^64^, a kind gift of Dr. Guus Wolswijk, NIN, Amsterdam, the Netherlands) and A2B5, an antibody against c-series gangliosides (kind gift of Dr. Thijs Lopez-Cardozo, Utrecht, the Netherlands), which are enriched at the surface of OPCs^65,66^ was performed at 4 °C. Non-specific antibody binding was blocked with 4% bovine serum albumin (BSA) for 10 minutes and cells were incubated with A2B5 (1:5 in 4% BSA) for 30 minutes. Cells were rinsed twice with PBS and incubated with appropriate FITC-conjugated antibody (1:50, Jackson ImmunoResearch). After washing twice with PBS, cells were fixed with 4% paraformaldehyde (PFA) in PBS for 20 minutes at room temperature (RT) and incubated for 15 minutes with 1 µg/ml DAPI (Sigma-Aldrich, cat. no. 32670) for nuclear counterstaining. For staining of internal components, PFA-fixed cells were permeabilized with 0.1% Triton X-100 (ki67, Olig2) for 30 minutes or ice-cold methanol (MBP) for 10 minutes. Non-specific antibody binding was blocked with 4% BSA for 30 minutes after which cells were incubated with either anti-ki67 (1 µg/ml; Abcam, cat. no. ab15580), anti-myelin basic protein (MBP, 1:250 in BSA; Serotec, cat. no. MCA409S) and/or anti-Olig2 (OLG lineage marker, 1:100 in BSA; Millipore, cat. no. AB9610) antibodies at RT. Cells were washed 3 times with PBS before the appropriate FITC-/TRITC-conjugated antibodies (1:50) were added together with 1 µg/ml DAPI for 30 minutes at RT. After washing with PBS, coverslips were mounted using Dako mounting medium (Dako, cat. no. S3025). Samples were analysed using a conventional immunofluorescence microscope (Leica DMI 6000 B with Leica Application Suite Advanced Fluorescence software) equipped with a 40× objective. In each independent experiment, approximately 150–250 cells were scored per condition.

### Morphology

Morphological analysis was performed as described by Langhammer and colleagues^26^. Briefly, A2B5-immunolabeled OPCs were photographed using a conventional immunofluorescence microscope (Leica DMI 6000 B), with 20× objective and photos were converted to 8-bit TIFF files. TIFF files were loaded in FIJI^67^ and cellular processes were traced using the NeuronJ plugin^68^. Traces were converted to SWC files by the Bonfire-program written for MATLAB^26^. SWC files were adapted in NeuronStudio^69^ after which Sholl-analysis and measurements of other morphological endpoints were performed by drawing concentric circles around the cell body with an incrementing radius of 6 µm. In each independent experiment 24–26 cells were analysed per condition. Mean values of each independent experiment were taken and plotted.

### Survival assay

OPCs were plated in PLL-coated 24-well plates (Nunc; Thermo Fisher Scientific, cat. no. 144530) in triplicate. OPC survival upon cytokine exposure was assessed by 3-(4,5-Dimethyl-2-thiazolyl)-2,5-diphenyl-2H-tetrazolium bromide (MTT; Sigma-Aldrich, cat. no. M2128)-reduction and lactate dehydrogenase (LDH; Roche, cat. no. 11644793001) assays. For the MTT-reduction assay, 500 μg/ml MTT was added to each well and left to incubate for 4 hours at 37 °C. Cells were resuspended in dimethyl sulfoxide and absorption was measured at 570 nm. LDH assays were performed according to manufacturer’s instructions on medium of cells analysed in the MTT-reduction assay and related to medium of lysed untreated cells.

### Migration assay

OPCs were plated at a density of 1 × 10^5^ on a PLL-coated porous membrane of a transwell insert with a pore size of 8 μm (Falcon, cat. no. 734–0053). A chemoattractive gradient was created by the addition of PDGF-AA (10 ng/ml) under the transwell insert. OPCs were allowed to migrate for 4 hours after which cells were fixed for 20 minutes in ice cold 5% acetic acid in ethanol. Cells were washed once with PBS. Cells on top of the transwell membrane were removed using a cotton swab, and nuclei of migrated cells were stained with 1 µg/ml DAPI. After washing thrice with PBS, the membranes were cut from the transwell insert and mounted in Dako mounting medium under a glass coverslip. Fluorescent images of the whole membrane were taken with the TissueFAXS fluorescent microscope. The average number of migrated cells per mm^2^ was calculated using Tissuequest 4.0 software. Total number of migrated cells was calculated from the total surface of the membrane and related to total plated cells after correction for non-adhering cells via the adhesion assay.

### Adhesion assay

Cells were plated at density of 1 × 10^5^ in PLL-coated wells in triplicate on a 96-well plate (Nunc; Thermo Fisher Scientific, cat. no. 167008) in 50 μl Sato medium. After one hour, PDGF-AA was added. After 4 hours, cells were fixed with ice cold methanol for 10 minutes. Cells were washed with PBS after which 0.2% crystal violet solution in ethanol was added for 10 minutes. Wells were then washed thrice with water and cells were dissolved in 1% sodium dodecyl sulphate. Absorption was measured at 570 nm after 30 minutes. Adhesion of gmOPCs was set to 1 for further analysis.

### qPCR analysis

For OPC maturation markers OPCs were plated at a density of 10^6^ cells in PLL-coated Petri dishes (Nunc; Thermo Fisher Scientific, cat. no. 172958). After 2 days, cells were gently scraped in PBS. For IFNγ/TNFα receptors mRNA was immediately isolated after shake-off, corresponding with the time point at which they were exposed to TNFα and/or IFNγ. mRNA was isolated using an mRNA-isolation kit (Isolate II RNA Micro Kit; Bioline, cat. no. BIO-52075) according to manufacturer’s instructions. 0.1 μg total RNA was reverse transcribed in the presence of oligo(dT)12–18 (Invitrogen, cat. no. 18418012) and dNTPs (Invitrogen, cat. no. 10297018) with M-MLV reverse transcriptase (Invitrogen, cat. no. 28025013) according to manufacturer’s instructions. Gene expression levels were measured by real-time quantitative RT-PCR using ABsolute QPCR SYBR Green Master Mix (Westburg, cat. no. AB-1163) in a Step-One Plus Real-Time PCR machine (Applied Biosystems). Each measurement was performed in triplicate and amplification data was processed using the LinRegPCR method^70,71^. Primer sequences are shown in Table 1. Relative expression to 2 housekeeping genes (*Eef1a1* and *Hmbs*) was calculated.

**Table 1:** Primer sequences used for RT-qPCR.

Gene	NCBI reference sequence	Forward primer	Reverse primer
*Bmp4*	NM_012827.2	TGAGGGATCTTTACCGGCTC	CTCCAGATGTTCTTCGTGATGG
*Cnp*	NM_012809.2	CAACAGGATGTGGTGAGGAG	CCTGCTCATTAAGCACCACC
*Eef1a1*	NM_175838.1	GATGGCCCCAAATTCTTGAAG	GGACCATGTCAACAATTGCAG
*Gpr17*	NM_001071777.1	CTTCTCTGGCAATCACTGGC	ACTTGACTGGGTGCACAATG
*Hes1*	NM_024360.3	GAAAGATAGCTCCCGGCATTC	GTACTTCCCCAACACGCTC
*Hes5*	NM_024383.1	TGAAGCACAGCAAAGCCTTC	ACGAGTAACCCTCGCTGTAG
*Hmbs*	NM_013168.2	CCGAGCCAAGCACCAGGAT	CTCCTTCCAGGTGCCTCAGA
*Id2*	NM_013060.3	CCAGAGACCTGGACAGAACC	GAATTCAGACGCCTGCAAGGAC
*Ifngr1*	NM_053783.1	CTAAGTCCTTGCTCTCTGTGG	GTCACTGTGGACAAGTGCTC
*Ifngr2*	NM_001108313.1	TACAGCTACGTCGATGGCTC	GCGCAGGAAGACTGTGTATG
*Itpr2*	NM_031046.3	GCTACACAACAACCGCAAAC	TAGTCCAGAAACCTCGGCTC
*Lpar1*	NM_053936.3	TGCCCTTTGGCCAGGCTTTC	AGTTTGGAGCGATGAAGAGGC
*Mbp*	NM_001025289.1	AGAACTACCCACTACGGCTC	GGTGTACGAGGTGTCACAATG
*Myrf*	NM_001170487.1	AGCCCTCCAATATAGACACCAG	TCAGGGAAGCAGAGGTCAG
*Neu4*	NM_001108234.1	CTATTGCTTTACGCTCCCTGG	CGGTTCTCTTGGGAACATGC
*Nkx6–2*	NM_001107558.2	AGAGCCAGGTGAAGGTGTG	TCTTTTTAGCCGACGCCATC
*Opalin*	NM_001017386.1	TGAGCCCATCGAGGAGACTG	TGTGACCTTCTTGAGCACCTC
*Pdgfra*	NM_012802.1	AAGATGCTCAAACCCACAGC	ACAATGTTCAGATGCGGTCC
*Sox6*	NM_001024751.1	ATGCCTCCGCTCATGATCCC	GGGGTAGTTATCACCTGGCTTG
*Sox9*	NM_080403.1	CAAGCTCTGGAGACTGCTG	GCCCATTCTTCACCGACTTC
*Tcf7l2*	NM_001191052.1	CATTTTCAATCCGGCAGCAC	CTTCCTGCTTGGACATCGAG
*Tnfrsf1a*	NM_013091.1	TGTCCCCAGGGAAAGTATGC	CCAAGTAGGTTCCTTTGTGGC
*Tnfrsf1b*	NM_130426.4	ATTGAACCAAGCATCACGGG	GCAGGAGGGCTTCTTTTTCC

### Statistical analysis

Data are expressed as mean ± standard error of the mean (SEM) for at least three independent experiments. When absolute values between two groups were compared (i.e., gmOPCs *vs* wmOPCs) statistical significance was assessed using a paired two-sided t-test and when more than two groups were compared a one-way ANOVA followed by a Tukey’s post-test was used. Statistical analysis was performed with a one-sample t-test when relative values of groups were compared by setting the untreated control values at 1 at each independent experiment. When relative values of two conditions were compared between gmOPCs and wmOPCs an unpaired two-sided t-test was used. When relative values of multiple treatment conditions were compared between gmOPCs and wmOPCs a one-way ANOVA with a Šidák post-test was used. Statistics were performed using GraphPad Prism 6.0. In all cases p-values of <0.05, <0.01, and <0.001 were considered significant and indicated with *, **, *** respectively.

### Data availability

All data generated during and/or analysed during the current study are available from the corresponding author on reasonable request.

## Electronic supplementary material

Supplementary information
