# Supplementary material for: Grey matter OPCs are less mature and less sensitive to IFNγ than white matter OPCs: consequences for remyelination
Source: Sci Rep. 2018 Feb 1;8:2113. doi: 10.1038/s41598-018-19934-6 (PMC5794790; doi:10.1038/s41598-018-19934-6)
Supplement: Supplementary file 1 — Supplementary information [file 41598_2018_19934_MOESM1_ESM.pdf]

Supplementary information

Grey matter OPCs are less mature and less sensitive to IFNγ than white matter OPCs: consequences for remyelination

Dennis H. Lentferink[1,‡], Jacomien M. Jongsma[1,‡], Inge Werkman[1], and Wia Baron[1,#]

[1] Department of Cell Biology, University Medical Center Groningen, University of Groningen, A. Deusinglaan 1, 9713 AV, the Netherlands

[‡] contributed equally to this work

[#] Corresponding author:

Wia Baron: Department of Cell Biology, University Medical Center Groningen, University of Groningen, A. Deusinglaan 1, 9713 AV Groningen, the Netherlands, tel.: +31 503616116, fax: +31 503616190, email: w.baron@umcg.nl

Primary cell cultures

The forebrains of 1-2 day old male and female Wistar rats were collected and cerebral hemispheres were separated. Olfactory bulbs were removed. The neonatal cortex [referred to as grey matter oligodendrocyte progenitor cells (gmOPCs)] and neonatal non-cortical parts [WM tracts including corpus callosum, mixed GM and WM tracts, including hippocampus and thalamus, and deep GM parts, including basal ganglia, referred to as white matter OPCs (wmOPCs)] of the forebrain were separated and meninges were removed. Cortices and non-cortices were minced and incubated with papain digestion mix [240 μg/ml L-cysteine (Sigma-Aldrich, cat. no. C7477), 40 μg/ml DNase I (Roche, cat. no. 10104159001), 30U papain from papaya latex (Sigma-Aldrich, cat. no. P3125) in MEM (Life Technologies, cat.no. 31095)] for 50 minutes at 37ºC. Enzymatic digestion was stopped by the addition of OVO [40 μg/ml DNase I (Sigma-Aldrich, cat. no. 10104159001), 1 mg/ml trypsin inhibitor (Sigma-Aldrich, cat. no. T6522), 50 μg/ml BSA (Sigma-Aldrich, cat. no. A4919) in L15 medium (Sigma-Aldrich, cat. no. L4386)] twice for three minutes. After gentle resuspension, the single cell suspension was plated onto poly-L-lysine (PLL; 5 μl/ml; Sigma-Aldrich, cat. no. P2636) coated flasks (Nunc T75; Thermo Fisher Scientific, cat. no. 153732) at a density of 1.25-1.5 cortices or 3-3.5 non-cortices per flask containing O2A medium [100U/ml penicillin and streptomycin (Invitrogen, cat. no. 15140), 4mM L-glutamine (Invitrogen, cat. no. 25030), 10% (v/v) foetal bovine serum (FBS, Capricorn Scientific, cat. no. FBS-12A) in DMEM (Life Technologies, 41965] and cultured for 12-14 days at 37ºC and 7.5% CO_2. Flasks were shaken for 1 hour at 150 rpm on an orbital shaker (New Brunswich Scientific, Innova 4000) after which detached microglia were removed. Flasks were then shaken overnight at 240 rpm and detached OPCs were collected and placed onto Petri dishes (Greiner Bio-One, cat. no. 633102) and incubated for 15 minutes at 37ºC for further purification via differential adhesion of microglia and astrocytes but not OPCs. The enriched OPC fraction contained 95-97% OPCs (Olig2-positive), less than 1% microglia (IB4-positive), 1-3% astrocytes (GFAP-positive) and less

than 1% neurons (TuJ1-positive). An additional overnight shake at 240 rpm yielded an enriched (>97%) astrocyte population.

Sato medium

DMEM containing 5 μg/ml bovine insulin (Sigma, cat. no. I1882), 50 μg/ml human holo-transferrin (Sigma-Aldrich, cat. no. T0665), 100 μg/ml bovine serum albumin fraction V (Sigma-Aldrich, cat. no. A3311), 62 ng/ml progesterone (Sigma-Aldrich, cat. no. P8783), 16 μg/ml putrescine (Sigma-Aldrich, cat. no. P5780), 5 ng/ml sodium selenite (Sigma-Aldrich, cat. no. S5261), 400 ng/ml T3 (Sigma-Aldrich, cat. no. T6397), 400 ng/ml T4 (Sigma-Aldrich, cat. no. T1775), 4 mM L-glutamine, 100U/ml penicillin and streptomycin and 27.5 μM 2-mercaptoethanol (BME; Sigma-Aldrich, cat. no. 21985).
